# Supplementary material for: TROP2‐Targeting Chimeras (TRTACs) for Tumor Cell‐Selective Membrane Protein Degradation and Enhanced Drug Delivery
Source: Adv Sci (Weinh). 2026 Sep 27:e77898. Online ahead of print. doi: 10.1002/advs.77898 (PMC13616387; doi:10.1002/advs.77898)
Supplement: Supplementary file 1 [file ADVS-9999-e77898-s001.docx]

Supporting Information

TROP2-targeting chimeras (TRTACs) for tumor cell-selective membrane protein degradation and enhanced drug delivery

Luping Chen,^†^ Xinying Fu,^†^ Wenqian Dong, Xiaobei Deng, Simin Chen, Fujun Wang, Jian Zhao, Shiqun Shao,* Liqiang Fan,* Jingyu Zhang,* and Lixin Zhang

L. Chen, X. Fu, W. Dong, S. Chen, J. Zhao, L. Fan, J. Zhang, Lixin Zhang

State Key Laboratory of Bioreactor Engineering, East China University of Science and Technology, Shanghai 200237, China

E-mail: fanglq@ecust.edu.cn (L. Fan), zhangjingyu@ecust.edu.cn (J. Zhang)

X. Deng, S. Shao

Zhejiang Key Laboratory of Smart Biomaterials and Center for Bionanoengineering, College of Chemical and Biological Engineering, Zhejiang University, Hangzhou, Zhejiang 310058, China

E-mail: shiqun.shao@zju.edu.cn (S. Shao)

F. Wang

New Drug R&D Center, Zhejiang Fonow Medicine Co. Ltd., Dongyang, Zhejiang 322100, China

F. Wang

Institute of Chinese Materia Medica, Shanghai University of Traditional Chinese Medicine, Shanghai, 201203, China

**Supporting Information text**

Protein sequence

11A4

MGEVQLVESGGGLVQAGGSLRLSCATSGITFMRYALGWYRQSPGKQREMVASINSGGTTNYADSVKGRFTISRDNAKNTVYLQMNSLKPEDTAVYYCNARWVKPQFIDNNYWGQGTQVTVSSLELEHHHHHH

11A4-cys

MGSSHHHHHHSSGLVPRGSHMEVQLVESGGGLVQAGGSLRLSCATSGITFMRYALGWYRQSPGKQREMVASINSGGTTNYADSVKGRFTISRDNAKNTVYLQMNSLKPEDTAVYYCNARWVKPQFIDNNYWGQGTQVTVSSLEGSGC

7D12-cys

MGSSHHHHHHSSGLVPRGSHMQVKLEESGGGSVQTGGSLRLTCAASGRTSRSYGMGWFRQAPGKEREFVSGISWRGDSTGYADSVKGRFTISRDNAKNTVDLQMNSLKPEDTAIYYCAAAAGSAWYGTLYEYDYWGQGTQVTVSSGSGC

7D12-11A4

MAMQVKLEESGGGSVQTGGSLRLTCAASGRTSRSYGMGWFRQAPGKEREFVSGISWRGDSTGYADSVKGRFTISRDNAKNTVDLQMNSLKPEDTAIYYCAAAAGSAWYGTLYEYDYWGQGTQVTVSSGSGGGGSGGGGSEVQLVESGGGLVQAGGSLRLSCATSGITFMRYALGWYRQSPGKQREMVASINSGGTTNYADSVKGRFTISRDNAKNTVYLQMNSLKPEDTAVYYCNARWVKPQFIDNNYWGQGTQVTVSSLELEHHHHHH

RLM(TF)-EGFP

MGRLMTQDCLQQSRKGGGGSGGGGSGGGGSRLMTQDCLQQSRKGGGGSGGGGSGGGGSRLMTQDCLQQSRKGGGGSGGGGSGSGGGGSVSKGEELFTGVVPILVELDGDVNGHKFSVSGEGEGDATYGKLTLKFICTTGKLPVPWPTLVTTLTYGVQCFSRYPDHMKQHDFFKSAMPEGYVQERTIFFKDDGNYKTRAEVKFEGDTLVNRIELKGIDFKEDGNILGHKLEYNYNSHNVYIMADKQKNGIKVNFKIRHNIEDGSVQLADHYQQNTPIGDGPVLLPDNHYLSTQSALSKDPNEKRDHMVLLEFVTAAGITLGMDELYKLEHHHHHH

TROP2^Nb^-EGFP

MGQVQLQESGGGLVQPGGSLRLSCAASGFTLDSYAIAWFRQAPGKEREGVSCIRSKDGSTYYADSVKGRFTISRDNAKNTVYLQMNSLKPEDTAVYYCAACDDDDEGGIIKMRSSSTVVWYYYYWGKGTQVTVSSGSGGGGSVSKGEELFTGVVPILVELDGDVNGHKFSVSGEGEGDATYGKLTLKFICTTGKLPVPWPTLVTTLTYGVQCFSRYPDHMKQHDFFKSAMPEGYVQERTIFFKDDGNYKTRAEVKFEGDTLVNRIELKGIDFKEDGNILGHKLEYNYNSHNVYIMADKQKNGIKVNFKIRHNIEDGSVQLADHYQQNTPIGDGPVLLPDNHYLSTQSALSKDPNEKRDHMVLLEFVTAAGITLGMDELYKLEHHHHHH

EGFP^Nb^-TROP2^Nb^

MGQVQLVESGGALVQPGGSLRLSCAASGFPVNRYSMRWYRQADTNNDGWIEGDELKEREWVAGMSSAGDRSSYEDSVKGRFTISRDDARNTVYLQMNSLKPEDTAVYYCNVNVGFEYWGQGTQVTVSSGSGGGGSGGGGSGGGGSQVQLQESGGGLVQPGGSLRLSCAASGFTLDSYAIAWFRQAPGKEREGVSCIRSKDGSTYYADSVKGRFTISRDNAKNTVYLQMNSLKPEDTAVYYCAACDDDDEGGIIKMRSSSTVVWYYYYWGKGTQVTVSSLEHHHHHH

11A4-EGFP

MGEVQLVESGGGLVQAGGSLRLSCATSGITFMRYALGWYRQSPGKQREMVASINSGGTTNYADSVKGRFTISRDNAKNTVYLQMNSLKPEDTAVYYCNARWVKPQFIDNNYWGQGTQVTVSSLEGGGGSGGGGSGSGGGGSVSKGEELFTGVVPILVELDGDVNGHKFSVSGEGEGDATYGKLTLKFICTTGKLPVPWPTLVTTLTYGVQCFSRYPDHMKQHDFFKSAMPEGYVQERTIFFKDDGNYKTRAEVKFEGDTLVNRIELKGIDFKEDGNILGHKLEYNYNSHNVYIMADKQKNGIKVNFKIRHNIEDGSVQLADHYQQNTPIGDGPVLLPDNHYLSTQSALSKDPNEKRDHMVLLEFVTAAGITLGMDELYKLEHHHHHH

7D12-RLM(TF)

MAMQVKLEESGGGSVQTGGSLRLTCAASGRTSRSYGMGWFRQAPGKEREFVSGISWRGDSTGYADSVKGRFTISRDNAKNTVDLQMNSLKPEDTAIYYCAAAAGSAWYGTLYEYDYWGQGTQVTVSSGSGGGGSGGGGSRLMTQDCLQQSRKGGGGSGGGGSGGGGSRLMTQDCLQQSRKGGGGSGGGGSGGGGSRLMTQDCLQQSRKLEHHHHHH

11A4-TROP2^Nb^-cys

MGSSHHHHHHSSGLVPRGSHMEVQLVESGGGLVQAGGSLRLSCATSGITFMRYALGWYRQSPGKQREMVASINSGGTTNYADSVKGRFTISRDNAKNTVYLQMNSLKPEDTAVYYCNARWVKPQFIDNNYWGQGTQVTVSSLEGSGGGGSGGGGSQVQLQESGGGLVQPGGSLRLSCAASGFTLDSYAIAWFRQAPGKEREGVSCIRSKDGSTYYADSVKGRFTISRDNAKNTVYLQMNSLKPEDTAVYYCAACDDDDEGGIIKMRSSSTVVWYYYYWGKGTQVTVSSGSGC

TROP2^Nb108^-EGFP

MGQVQLQESGGGLVQPGGSLRLSCAASGRIFSSFAMAWFRQAPGKEREFVTAITWSGGSTYYADSVKGRFTISRDNAKNTVYLQMNSLKPEDTAVYYCAAADDEGLSTVVYYYWGQGTQVTVSSGGGGSGGGGSGSGGGGSVSKGEELFTGVVPILVELDGDVNGHKFSVSGEGEGDATYGKLTLKFICTTGKLPVPWPTLVTTLTYGVQCFSRYPDHMKQHDFFKSAMPEGYVQERTIFFKDDGNYKTRAEVKFEGDTLVNRIELKGIDFKEDGNILGHKLEYNYNSHNVYIMADKQKNGIKVNFKIRHNIEDGSVQLADHYQQNTPIGDGPVLLPDNHYLSTQSALSKDPNEKRDHMVLLEFVTAAGITLGMDELYKLEHHHHHH

CLND18.2^Nb^-EGFP

MGQVQLVESGGGLVQPGGSLRLSCAASGVDISSDVMAWYRQAPGKGLEFVSGLTRGGSINYADSVKGRFTISRDNSKNTLYLQMNSLRAEDTAVYYCNAEIYTGTFYPRSYWGQGTLVTVSGGGGSGGGGSGSGGGGSVSKGEELFTGVVPILVELDGDVNGHKFSVSGEGEGDATYGKLTLKFICTTGKLPVPWPTLVTTLTYGVQCFSRYPDHMKQHDFFKSAMPEGYVQERTIFFKDDGNYKTRAEVKFEGDTLVNRIELKGIDFKEDGNILGHKLEYNYNSHNVYIMADKQKNGIKVNFKIRHNIEDGSVQLADHYQQNTPIGDGPVLLPDNHYLSTQSALSKDPNEKRDHMVLLEFVTAAGITLGMDELYKLEHHHHHH

C7(FRα)-EGFP

MGMHTAPGWGYRLSGGGGSGGGGSGGGGSMHTAPGWGYRLSGGGGSGGGGSGGGGSMHTAPGWGYRLSGGGGSGGGGSGSGGGGSVSKGEELFTGVVPILVELDGDVNGHKFSVSGEGEGDATYGKLTLKFICTTGKLPVPWPTLVTTLTYGVQCFSRYPDHMKQHDFFKSAMPEGYVQERTIFFKDDGNYKTRAEVKFEGDTLVNRIELKGIDFKEDGNILGHKLEYNYNSHNVYIMADKQKNGIKVNFKIRHNIEDGSVQLADHYQQNTPIGDGPVLLPDNHYLSTQSALSKDPNEKRDHMVLLEFVTAAGITLGMDELYKLEHHHHHH

CD44^Nb^-EGFP

MGMQVQLVESGGGSVQAGGSLRLSCTASGGSEYSYSTFSLGWFRQAPGQEREAVAAIASMGGLTYYADSVKGRFTISRDNAKNTVTLQMNNLKPEDTAIYYCAARNAGGRFRPSAAGGYNYWGQGTLVTVSSGGGGSGGGGSGSGGGGSVSKGEELFTGVVPILVELDGDVNGHKFSVSGEGEGDATYGKLTLKFICTTGKLPVPWPTLVTTLTYGVQCFSRYPDHMKQHDFFKSAMPEGYVQERTIFFKDDGNYKTRAEVKFEGDTLVNRIELKGIDFKEDGNILGHKLEYNYNSHNVYIMADKQKNGIKVNFKIRHNIEDGSVQLADHYQQNTPIGDGPVLLPDNHYLSTQSALSKDPNEKRDHMVLLEFVTAAGITLGMDELYKLEHHHHHH

IGF2-11A4

MGLCGGELVDTLQFVCGDRGFYFSRPASRVSRRSRGIVEECCFRSCDLALLETYCATPAKSEEFGSGGGGSGGGGSEVQLVESGGGLVQAGGSLRLSCATSGITFMRYALGWYRQSPGKQREMVASINSGGTTNYADSVKGRFTISRDNAKNTVYLQMNSLKPEDTAVYYCNARWVKPQFIDNNYWGQGTQVTVSSLELEHHHHHH

7D12-TROP2^Nb108^

MAMQVKLEESGGGSVQTGGSLRLTCAASGRTSRSYGMGWFRQAPGKEREFVSGISWRGDSTGYADSVKGRFTISRDNAKNTVDLQMNSLKPEDTAIYYCAAAAGSAWYGTLYEYDYWGQGTQVTVSSGSGGGGSGGGGSQVQLQESGGGLVQPGGSLRLSCAASGRIFSSFAMAWFRQAPGKEREFVTAITWSGGSTYYADSVKGRFTISRDNAKNTVYLQMNSLKPEDTAVYYCAAADDEGLSTVVYYYWGQGTQVTVSSLEHHHHHH

7D12-CD44^Nb^

MAMQVKLEESGGGSVQTGGSLRLTCAASGRTSRSYGMGWFRQAPGKEREFVSGISWRGDSTGYADSVKGRFTISRDNAKNTVDLQMNSLKPEDTAIYYCAAAAGSAWYGTLYEYDYWGQGTQVTVSSGSGGGGSGGGGSMQVQLVESGGGSVQAGGSLRLSCTASGGSEYSYSTFSLGWFRQAPGQEREAVAAIASMGGLTYYADSVKGRFTISRDNAKNTVTLQMNNLKPEDTAIYYCAARNAGGRFRPSAAGGYNYWGQGTLVTVSSLEHHHHHH

TROP2^Nb^-cys

MGSSHHHHHHSSGLVPRGSHMQVQLQESGGGLVQPGGSLRLSCAASGFTLDSYAIAWFRQAPGKEREGVSCIRSKDGSTYYADSVKGRFTISRDNAKNTVYLQMNSLKPEDTAVYYCAACDDDDEGGIIKMRSSSTVVWYYYYWGKGTQVTVSSGSGC

11A4-TROP2^Nb^

MGEVQLVESGGGLVQAGGSLRLSCATSGITFMRYALGWYRQSPGKQREMVASINSGGTTNYADSVKGRFTISRDNAKNTVYLQMNSLKPEDTAVYYCNARWVKPQFIDNNYWGQGTQVTVSSLEGSGGGGSGGGGSGGGGSQVQLQESGGGLVQPGGSLRLSCAASGFTLDSYAIAWFRQAPGKEREGVSCIRSKDGSTYYADSVKGRFTISRDNAKNTVYLQMNSLKPEDTAVYYCAACDDDDEGGIIKMRSSSTVVWYYYYWGKGTQVTVSSLEHHHHHH

7D12-TROP2^Nb^-cys

MGSSHHHHHHSSGLVPRGSHMQVKLEESGGGSVQTGGSLRLTCAASGRTSRSYGMGWFRQAPGKEREFVSGISWRGDSTGYADSVKGRFTISRDNAKNTVDLQMNSLKPEDTAIYYCAAAAGSAWYGTLYEYDYWGQGTQVTVSSGSGGGGSGGGGSQVQLQESGGGLVQPGGSLRLSCAASGFTLDSYAIAWFRQAPGKEREGVSCIRSKDGSTYYADSVKGRFTISRDNAKNTVYLQMNSLKPEDTAVYYCAACDDDDEGGIIKMRSSSTVVWYYYYWGKGTQVTVSSGSGC

7D12-TROP2^Nb^

MAMQVKLEESGGGSVQTGGSLRLTCAASGRTSRSYGMGWFRQAPGKEREFVSGISWRGDSTGYADSVKGRFTISRDNAKNTVDLQMNSLKPEDTAIYYCAAAAGSAWYGTLYEYDYWGQGTQVTVSSGSGGGGSGGGGSQVQLQESGGGLVQPGGSLRLSCAASGFTLDSYAIAWFRQAPGKEREGVSCIRSKDGSTYYADSVKGRFTISRDNAKNTVYLQMNSLKPEDTAVYYCAACDDDDEGGIIKMRSSSTVVWYYYYWGKGTQVTVSSLEHHHHHH

KN035-TROP2^Nb^

MGTGQVQLQESGGGLVQPGGSLRLSCAASGKMSSRRCMAWFRQAPGKERERVAKLLTTSGSTYLADSVKGRFTISQNNAKSTVYLQMNSLKPEDTAMYYCAADSFEDPTCTLVTSSGAFQYWGQGTQVTVSSGSMDPGGSGSGGGGSGGGGSQVQLQESGGGLVQPGGSLRLSCAASGFTLDSYAIAWFRQAPGKEREGVSCIRSKDGSTYYADSVKGRFTISRDNAKNTVYLQMNSLKPEDTAVYYCAACDDDDEGGIIKMRSSSTVVWYYYYWGKGTQVTVSSLEHHHHHH

7D12-CLDN18.2^Nb^

MAMQVKLEESGGGSVQTGGSLRLTCAASGRTSRSYGMGWFRQAPGKEREFVSGISWRGDSTGYADSVKGRFTISRDNAKNTVDLQMNSLKPEDTAIYYCAAAAGSAWYGTLYEYDYWGQGTQVTVSSGSGGGGSGGGGSQVQLVESGGGLVQPGGSLRLSCAASGVDISSDVMAWYRQAPGKGLEFVSGLTRGGSINYADSVKGRFTISRDNSKNTLYLQMNSLRAEDTAVYYCNAEIYTGTFYPRSYWGQGTLVTVSLEHHHHHH

7D12-EGFP

MAMQVKLEESGGGSVQTGGSLRLTCAASGRTSRSYGMGWFRQAPGKEREFVSGISWRGDSTGYADSVKGRFTISRDNAKNTVDLQMNSLKPEDTAIYYCAAAAGSAWYGTLYEYDYWGQGTQVTVSSGSGGGGSVSKGEELFTGVVPILVELDGDVNGHKFSVSGEGEGDATYGKLTLKFICTTGKLPVPWPTLVTTLTYGVQCFSRYPDHMKQHDFFKSAMPEGYVQERTIFFKDDGNYKTRAEVKFEGDTLVNRIELKGIDFKEDGNILGHKLEYNYNSHNVYIMADKQKNGIKVNFKIRHNIEDGSVQLADHYQQNTPIGDGPVLLPDNHYLSTQSALSKDPNEKRDHMVLLEFVTAAGITLGMDELYKLEHHHHHH

7D12-CD44^binder^

MAMQVKLEESGGGSVQTGGSLRLTCAASGRTSRSYGMGWFRQAPGKEREFVSGISWRGDSTGYADSVKGRFTISRDNAKNTVDLQMNSLKPEDTAIYYCAAAAGSAWYGTLYEYDYWGQGTQVTVSSGSGGGGSGGGGSGGGGSDTYCFDTYCFDTYCFLEHHHHHH*

IGF2-7D12

MGLCGGELVDTLQFVCGDRGFYFSRPASRVSRRSRGIVEECCFRSCDLALLETYCATPAKSEEFGSGGGGSGGGGSQVKLEESGGGSVQTGGSLRLTCAASGRTSRSYGMGWFRQAPGKEREFVSGISWRGDSTGYADSVKGRFTISRDNAKNTVDLQMNSLKPEDTAIYYCAAAAGSAWYGTLYEYDYWGQGTQVTVSSLEHHHHHH

IGF2-KN035

MGLCGGELVDTLQFVCGDRGFYFSRPASRVSRRSRGIVEECCFRSCDLALLETYCATPAKSEEFGSGGGGSGGGGSGGGGSTGQVQLQESGGGLVQPGGSLRLSCAASGKMSSRRCMAWFRQAPGKERERVAKLLTTSGSTYLADSVKGRFTISQNNAKSTVYLQMNSLKPEDTAMYYCAADSFEDPTCTLVTSSGAFQYWGQGTQVTVSSGSMDPGGSLEHHHHHH

7D12-TROP2^Nb108^

MAMQVKLEESGGGSVQTGGSLRLTCAASGRTSRSYGMGWFRQAPGKEREFVSGISWRGDSTGYADSVKGRFTISRDNAKNTVDLQMNSLKPEDTAIYYCAAAAGSAWYGTLYEYDYWGQGTQVTVSSGSGGGGSGGGGSQVQLQESGGGLVQPGGSLRLSCAASGRIFSSFAMAWFRQAPGKEREFVTAITWSGGSTYYADSVKGRFTISRDNAKNTVYLQMNSLKPEDTAVYYCAAADDEGLSTVVYYYWGQGTQVTVSSLEHHHHHH

7D12-TROP2^VNAR-5G8^

MAMQVKLEESGGGSVQTGGSLRLTCAASGRTSRSYGMGWFRQAPGKEREFVSGISWRGDSTGYADSVKGRFTISRDNAKNTVDLQMNSLKPEDTAIYYCAAAAGSAWYGTLYEYDYWGQGTQVTVSSGSGGGGSGGGGSQWVEQTPTTTTKEAGESLTINCVLRDSSCPLASTYWYFTKKGATKKESLSNGGRYAETVNKASKSFSLRISDLRVEDSGTYHCKAVNSWTNCAPLERYYEGGGTILSVKPAALEHHHHHH

TROP2^VNAR-5G8^-EGFP

MGQWVEQTPTTTTKEAGESLTINCVLRDSSCPLASTYWYFTKKGATKKESLSNGGRYAETVNKASKSFSLRISDLRVEDSGTYHCKAVNSWTNCAPLERYYEGGGTILSVKPAAGGGGSGGGGSGSGGGGSVSKGEELFTGVVPILVELDGDVNGHKFSVSGEGEGDATYGKLTLKFICTTGKLPVPWPTLVTTLTYGVQCFSRYPDHMKQHDFFKSAMPEGYVQERTIFFKDDGNYKTRAEVKFEGDTLVNRIELKGIDFKEDGNILGHKLEYNYNSHNVYIMADKQKNGIKVNFKIRHNIEDGSVQLADHYQQNTPIGDGPVLLPDNHYLSTQSALSKDPNEKRDHMVLLEFVTAAGITLGMDELYKLEHHHHHH

11A4-TROP2^Nb^-EGFP

MGSSHHHHHHSSGLVPRGSHMEVQLVESGGGLVQAGGSLRLSCATSGITFMRYALGWYRQSPGKQREMVASINSGGTTNYADSVKGRFTISRDNAKNTVYLQMNSLKPEDTAVYYCNARWVKPQFIDNNYWGQGTQVTVSSLEGGGGSGGGGSGGGGSQVQLQESGGGLVQPGGSLRLSCAASGFTLDSYAIAWFRQAPGKEREGVSCIRSKDGSTYYADSVKGRFTISRDNAKNTVYLQMNSLKPEDTAVYYCAACDDDDEGGIIKMRSSSTVVWYYYYWGKGTQVTVSSGGGGSGGGGSGSGGGGSVSKGEELFTGVVPILVELDGDVNGHKFSVSGEGEGDATYGKLTLKFICTTGKLPVPWPTLVTTLTYGVQCFSRYPDHMKQHDFFKSAMPEGYVQERTIFFKDDGNYKTRAEVKFEGDTLVNRIELKGIDFKEDGNILGHKLEYNYNSHNVYIMADKQKNGIKVNFKIRHNIEDGSVQLADHYQQNTPIGDGPVLLPDNHYLSTQSALSKDPNEKRDHMVLLEFVTAAGITLGMDELYK

Recombinant human TROP2 protein

HTAAQDNCTCPTNKMTVCSPDGPGGRCQCRALGSGMAVDCSTLTSKCLLLKARMSAPKNARTLVRPSEHALVDNDGLYDPDCDPEGRFKARQCNQTSVCWCVNSVGVRRTDKGDLSLRCDELVRTHHILIDLRHRPTAGAFNHSDLDAELRRLFRERYRLHPKFVAAVHYEQPTIQIELRQNTSQKAAGDVDIGDAAYYFERDIKGESLFQGRGGLDLRVRGEPLQVERTLIYYLDEIPPKFSMKRLT

**Figure S1. Identification of TROP2 as an effective LTR with high clinical relevance.** (**A**) Constructs of nanobody-based heterobifunctional chimeras designed to recruit TROP2, HER2, CD44, CLDN18.2, FRα, or TF to EGFR. (**B**) Constructs of EGFP-fused nanobodies or binding peptides as probes against EGFR, TROP2, HER2, CD44, CLDN18.2, FRα, or TF. (**C**) Flow cytometry analysis of TROP2, CD44, FRα, TF, CLDN18.2, or HER2 expression on the surface of HeLa or SKBR3 cells following incubation with indicated protein probes. (**D**) Flow cytometry analysis of cell-surface EGFR levels in SKBR3 cells treated with 500 nM of 7D12, 7D12-CD44^Nb^, 7D12-TF^Binder^, 7D12-FRα^Binder^, 7D12-HER2^Nb^, 7D12-CLDN18.2^Nb^, or 7D12-TROP2^Nb^ for 24 h. (**E**) Western blot analysis of EGFR levels in SKBR3 cells treated with 100 nM of 7D12, 7D12-CD44^Nb^, 7D12-TF^Binder^, 7D12-FRα^Binder^, 7D12-HER2^Nb^, 7D12-CLDN18.2^Nb^, or 7D12-TROP2^Nb^ for 24 h. (**F**) Kaplan–Meier analysis of correlation between TACSTD2 expression and overall survival in patients with CESC (cervical squamous cell carcinoma), PAAD (pancreatic adenocarcinoma), LUAD (lung adenocarcinoma), or OV (ovarian serous cystadenocarcinoma). (**G**) Flow cytometry analysis of cells incubated with 1 μM of TROP2^Nb^-EGFP for 3, 6, or 9 h, then treated with or without trypan blue. (**H**) Flow cytometry analysis and quantification of cell-surface EGFR levels in HeLa cells treated with 100 nM of 7D12-TROP2^Nb^ for 24 h in the presence or absence of bafilomycin A1. (**I and J**) Western blot analysis of EGFR protein levels in HeLa cells treated with 100 nM of 7D12-TROP2^Nb^ for 24 h in the presence or absence of 10 μM of MG132 (**I**) or in the presence or absence of 100 nM of TROP2^Nb^ (**J**). (**K**) Western blot detection of HER2 and TROP2 co-immunoprecipitated with 11A4-TROP2^Nb^-EGFP from cell lysates using anti-EGFP nanobody-conjugated agarose beads. Data are presented as mean ± standard deviation (SD) (n = 3 biologically independent replicates) when relevant. *P* values were determined by one-way analysis of variance (ANOVA) with Turkey’s *post hoc* test. **P* < 0.05, ***P* < 0.01, ****P* < 0.001, *****P* < 0.0001.

**Figure S2. 7D12-TROP2^Nb^-induced EGFR internalization and degradation.** (**A** and **B**) Flow cytometry analysis of cell-surface EGFR levels in HeLa cells treated for 24 h with 100 nM of 7D12, TROP2^Nb^, 7D12 + TROP2^Nb^, 7D12-IGF2, gefitinib, or 7D12-TROP2^Nb^ (**A**) or with serial concentrations of 7D12-TROP2^Nb^ (**B**). (**C**) Heat map of TACSTD2 and EGFR expression in BxPC-3, HeLa, MDA-MB-231, NCI-H1975, NCI-N87, and SK-BR-3 cells. Data were obtained from the Cancer Cell Line Encyclopedia. (**D**) Pearson correlation between TROP2 expression and the DC_50_ for EGFR degradation across six cancer cell lines (BXPC-3, HeLa, MDA-MB-231, NCI-H1975, NCI-N87, and SKBR3). The line represents the linear regression fit with the 95% confidence interval. Pearson’s *r*, coefficient of determination (*R*^2^), *P* value, and sample size (*n*) are indicated. (**E**) MTT assay of MRC-5, HUVEC, MCF-10A, and BEAS-2B cells treated with serial concentrations of 7D12-TROP2^Nb^. (**F**) Flow cytometry analysis of cell-surface EGFR levels in HeLa cells treated with 500 nM of 7D12, 7D12-TROP2^NbV-5^, 7D12-TROP2^Nb108^, or 7D12-TROP2^Nb^ for 24 h. (**G**) MTT assay of HeLa cells treated with serial concentrations of 7D12, 7D12-TROP2^NbV-5^, 7D12-TROP2^Nb108^, or 7D12-TROP2^Nb^ for 7 d. (**H**) Molecular docking of TROP2 with TROP2^Nb^, TROP2^NbV-5^, or TROP2^Nb108^. (**I**) ELISA analysis of TROP2^Nb^-7D12, TROP2^Nb^-EGFP, TROP2^NbV-5^-EGFP, TROP2^Nb108^-EGFP, or EGFP binding to TROP2. Data are presented as mean ± SD (n = 3 biologically independent replicates) when relevant. *P* values were determined by one-way ANOVA with Turkey’s *post hoc* test. ****P* < 0.001, *****P* < 0.0001.

**Figure S3. 7D12-TROP2^Nb^-mediated EGFR degradation in 3D tumor spheroids and assessment of *in vivo* safety.** (**A**) Bright field images of HeLa, H1975, and SKBR3 cells in 2D and 3D culture. Scale bar: 500 μm. (**B**) Western blot analysis and quantification of EGFR levels in HeLa, H1975, and SKBR3 spheroids treated with different concentrations of 7D12-TROP2^Nb^ for 24 h (n = 3 biologically independent replicates). (**C**) Haematoxylin and eosin (H&E) staining of major organs, including heart, liver, spleen, lung, and kidney, collected from mice treated with PBS, 7D12-IGF2, or 7D12-TROP2^Nb^. Scale bar: 200 μm. Data are presented as mean ± SD when relevant. *P* values were determined by one-way ANOVA with Turkey’s *post hoc* test. ns, no significance; **P* < 0.05; ***P* < 0.01; ****P* < 0.001.

**Figure S4. TRTAC-mediated degradation of PD-L1 and HER2.** (**A**) Western blot analysis and quantification of PD-L1 levels in MDA-MB-231 cells treated for 24 h with serial concentrations of KN035-TROP2^Nb^, IGF2-KN035, or KN035. (**B** and **C**) Western blot analysis and quantification of HER2 levels in SKBR3 cells (**B**) or NCI-N87 cells (**C**) treated for 24 h with serial concentrations of 11A4-TROP2^Nb^. (**D**) Western blot analysis and quantification of PD-L1 levels in MDA-MB-231 cells treated with 100 nM of KN035-TROP2^Nb^ for 24 h in the presence or absence of bafilomycin A1 (50 nM). (**E**) Western blot analysis and quantification of HER2 levels in SKBR3 cells treated with 100 nM of 11A4-TROP2^Nb^ for 24 h in the presence or absence of bafilomycin A1. Data are presented as mean ± SD (n = 3 biologically independent replicates) when relevant. *P* values were determined by one-way ANOVA with Turkey’s *post hoc* test. ns, no significance; **P* < 0.05; ***P* < 0.01; ****P* < 0.001; *****P* < 0.0001.

**Figure S5. Characterization of TRTAC-DCs.** (**A**) Synthesis of 11A4-TROP2^Nb^-MMAE. (**B** and **C**) MTT assay of HeLa cells treated for 72 h with serial concentrations of MMAE or 11A4-TROP2^Nb^-MMAE (**B**) or with serial concentrations of MMAE, 7D12-MMAE, or 7D12-TROP2^Nb^-MMAE (**C**). (**D-G**) MTT assay of A431 (**D**), MDA-MB-231 (**E**), SKBR3 (**F**), or HUVEC (**G**) cells treated with serial concentrations of MMAE or 7D12-TROP2^Nb^-MMAE for 72 h. (**H**) Schematic illustration of establishment of gefitinib-resistant PC-9/GR cell strains. Gefitinib-sensitive PC-9 cells were cultured with stepwise increasing concentrations of gefitinib for generating resistant PC-9/GR cells. (**I** and **J**) MTT assay of PC-9 (**I**) or PC-9/GR (**J**) cells treated with serial concentrations of gefitinib, 7D12-TROP2^Nb^-MMAE, or free MMAE for 72 h. (**K**) Synthesis of 11A4-TROP2^Nb^-DOX. (**L** and **M**) MTT assay of SKBR3 (**L**) or HeLa (**M**) cells treated with serial concentrations of 11A4-TROP2^Nb^-DOX or indicated controls for 72 h. Data are presented as mean ± SD (n = 3 biologically independent replicates).

**Figure S6. Assessment of *in vivo* safety of 11A4- TROP2^Nb^-MMAE.** H&E staining of major organs, including heart, liver, spleen, lung, and kidney, collected from mice treated with PBS, TROP2^Nb^-MMAE, 11A4-MMAE or 11A4- TROP2^Nb^-MMAE. Scale bar: 200 μm.

**Figure S7. Plasmid design for expression of EGFP-fused nanobodies or binding peptides against membrane receptors.**

**Figure S8. SDS-PAGE of purified proteins.** Lane 1: Marker; Lane 2: TF^Binder^-EGFP; Lane 3: FRα^Binder^-EGFP; Lane 4: CLDN^18.2Nb^-EGFP; Lane 5: TROP2^Nb^-EGFP; Lane 6: CD44^Nb^-EGFP; Lane 7: HER2^Nb^-EGFP; Lane 8: 7D12-EGFP.

**Figure S9. Plasmid design for expression of 7D12–ligand fusion proteins**

**Figure S10. SDS-PAGE of purified proteins.** Lane 1: Marker; Lane 2: 7D12-TF^Binder^; Lane 3: 7D12-FRα^Binder^; Lane 4: 7D12-CLDN^18.2Nb^; Lane 5: 7D12-TROP2^Nb^; Lane 6: 7D12-CD44^Nb^; Lane 7: 7D12-HER2^Nb^; Lane 8: 7D12- CD44^Nb^.

**Figure S11. Plasmid design for expression of 7D12-TROP2^Nb^, 7D12-TROP2^Nb108^, 7D12-TROP2^NbV-5^, and IGF2-7D12.**

**Figure S12. SDS-PAGE of purified proteins.** Lane 1: Marker; Lane 2: 7D12-TROP2^Nb^; Lane 3: 7D12-TROP2^Nb108^; Lane 4: 7D12-TROP2^NbV-5^; Lane 5: 7D12; Lane 6: TROP2^Nb^; Lane 8: 7D12-IGF2.

**Figure S13. Plasmid design for expression of HER2- and PD-L1-targeted TRTACs and corresponding controls.**

**Figure S14. SDS-PAGE of purified proteins.** Lane 1: Marker; Lane 2: KN035; Lane 3: TROP2^Nb^; Lane 4: KN035-IGF2; Lane 5: KN035-TROP2^Nb^; Lane 6: 11A4; Lane 7: 11A4-IGF2; Lane 8: 11A4-TROP2^Nb^.

**Figure S15. Plasmid design for expression of EGFP-targeted TRTAC and soluble EGFP.**

**Figure S16. SDS-PAGE of purified proteins.** Lane 1: Marker; Lane 2: EGFP; Lane 3: EGFP^Nb^-TROP2^Nb^.

**Figure S17. SDS-PAGE of purified proteins.** Lane 1: Marker; Lane 2: 11A4-TROP2^Nb^; Lane 3: 11A4-TROP2^Nb^-MMAE; Lane 4: 11A4; Lane 5: 11A4-MMAE; Lane 6: TROP2^Nb^; Lane 7: TROP2^Nb^-MMAE.

**Figure S18.** **Liquid chromatography-mass spectrometry (LC-MS) of 11A4-TROP2^Nb^ and 11A4-TROP2^Nb^-MMAE.**

**Figure S19. HPLC analysis of 11A4-TROP2^Nb^-MMAE and MMAE confirming the removal of free MMAE.**

**Figure S20. Gating strategy for flow cytometry.**

**WB raw data**

**Figure 1D**

**Figure 1H**

**Figure S1E**

**Figure S1I**

**Figure S1J**

**Figure S1K**

**Figure 2A**

**Figure 2B**

**Figure 2C 7D12**

**Figure 2C 7D12-IGF2**

**Figure 2C 7D12-TROP2^Nb^**

**Figure 2D SKBR3 cells**

**Figure 2D MDA-MB-231 cells**

**Figure 2D NCI-N87 cells**

**Figure 2D BxPC-3 cells**

**Figure 2D NCI-H1975 cells**

**Figure 2E MRC-5 cells**

**Figure 2E HUVEC cells**

**Figure S2E MCF-10A cells**

**Figure S2E Beas-2b cells**

**Figure S3B HeLa cells**

**Figure S3B H1975 cells**

**Figure S3B SKBR3 cells**

**Figure 4C**

**Figure 4E**

**Figure S4A**

**Figure S4A**

**Figure S4A**

**Figure S4B**

**Figure S4C**

**Figure S4D**

**Figure S4E**
